# Supplementary material for: An experimental test of whether financial incentives constitute undue inducement in decision-making
Source: Nat Hum Behav. 2024 Mar 8;8(5):835–45. doi: 10.1038/s41562-024-01817-8 (PMC11132984; doi:10.1038/s41562-024-01817-8)
Supplement: Supplementary file 1 — Supplementary Tables A.1–A.4, B.1–B.3 and C.1; Figs. B.1, C.1, C.2 and D.1; and text. [file 41562_2024_1817_MOESM1_ESM.pdf]

nature human behaviour

Article https://doi.org/10.1038/s41562-024-01817-8

An experimental test of whether financial incentives constitute undue inducement in decision-making

In the format provided by the authors and unedited

Table of Contents

A SI Experiment 1 **1**
A.1 Randomization check . . . 1
A.2 Reservation prices . . . 2
A.3 Welfare analysis by species . . . 2

B SI Experiment 2 **6**

C SI Experiment 3 **13**
C.1 Design . . . 13
C.2 Analysis . . . 15

D SI Theory **17**

References **24**

A SI Experiment 1

A.1 Randomization check

The four treatments are balanced across demographic characteristics. Table A.1 displays summary statistics of these variables by treatment. For each variable, the table reports the p-value of an F-test for differences in the variable's mean value across treatments. Of 24 tests conducted, one is significant at the 5% level.

Table A.1: Summary statistics and randomization check.

	(1)	(2)	(3)	(4)	(5)
Treatment condition					
Incentive	\$30	\$3	\$30	\$3	
Video	Yes	Yes	No	No	
Variable			Mean		p-value
Male	0.55	0.53	0.54	0.54	0.997
Age	21.43	22.01	21.37	21.30	0.337
Ethnicity					
African-American	0.05	0.06	0.07	0.07	0.701
Caucasian	0.57	0.51	0.59	0.56	0.280
East Asian	0.19	0.26	0.19	0.23	0.224
Hispanic	0.07	0.08	0.04	0.04	0.987
Indian	0.03	0.04	0.04	0.07	0.557
Other	0.08	0.05	0.07	0.04	0.703
Monthly spending in USD	251.72	301.40	289.07	288.42	0.443
Year of study	3.50	3.60	3.61	3.47	0.317
Graduate student	0.13	0.15	0.13	0.05	0.068
Field of study					
Arts and humanities	0.16	0.09	0.13	0.11	0.042
Business or economics	0.27	0.36	0.34	0.43	0.086
Engineering	0.20	0.16	0.11	0.12	0.490
Science	0.21	0.23	0.27	0.23	0.466
Social science (excluding business and economics)	0.17	0.17	0.15	0.11	0.593
Political orientation	0.50	0.32	0.27	0.09	0.081
Raven's score	14.77	14.76	14.69	14.68	1.000
CRT score	3.76	3.80	3.50	3.22	0.078
Experience with insects as food (1 = Yes, 0 = No)					
Has intentionally eaten insects before	0.19	0.22	0.19	0.20	0.715
Grown up in culture that practices entomophagy	0.15	0.14	0.13	0.15	0.925
Grown up eating mostly western foods	0.81	0.73	0.82	0.78	0.057
Had a pet that feeds on store-bought insects	0.25	0.25	0.21	0.26	0.684
Knew that this study concerns insect eating	0.20	0.30	0.26	0.29	0.231

***Notes*:** p-values in column (5) correspond to an $F(3, N-4)$-test of the joint significance of a regression of the indicated variable on treatment dummies where N is the number of non-missing observations for each listed characteristic. Year of study only includes undergraduate students. Political orientation is measured on a scale of -2 (conservative) to 2 (liberal). Raven's score is measured on a scale of 0 to 24. CRT score indicates the number of correct answers (out of 6) on an extended version of the CRT test [1].

A.2 Reservation prices

Table A.2 displays mean reservation prices. Because elicited reservation prices are interval coded and censored above at $60, I estimate these means using interval regression which accounts for both issues. These means represent substantial subject heterogeneity. Population standard deviations exceed $20 for each species both before and after the handout of the insects.

Table A.2: Reservation prices for eating insects

	(1)	(2)	(3)	(4)	(5)	(6)
VARIABLES	Reservation price					
Species	All	2 house crickets	5 meal-worms	3 silkworm pupae	2 mole crickets	2 field crickets
Levels						
Before	23.976***	20.283***	28.084***	23.678***	24.039***	23.769***
	(0.847)	(0.844)	(0.927)	(0.889)	(0.901)	(0.953)
After	26.164***	23.957***	27.447***	26.210***	30.719***	21.995***
	(0.874)	(0.919)	(0.940)	(0.922)	(0.954)	(0.928)
Difference	2.188***	3.674***	-0.637	2.532***	6.680***	-1.774***
	(0.342)	(0.484)	(0.494)	(0.470)	(0.526)	(0.520)
Observations	6,552	1,342	1,342	1,342	1,342	1,184
Subjects	671	671	671	671	671	592

Notes: Mean reservation prices, estimated by interval regression. Standard errors in parentheses, clustered by subject. The number of observations in column 6 is smaller because the 79 Stanford subjects who first participated in this experiment were not given any decisions regarding field crickets.

Column 1 of Table A.3 shows the ethnic makeup of the subject population. Columns 2 to 3 examine whether subjects of different ethnicities differ in terms of takeup of the promised offer, as well as reservation prices before and after the handout of the insects, respectively. The only statistically significant effect concerns Indians who have substantially higher reservation prices.

A.3 Welfare analysis by species

The analysis of welfare effects in Section 2.1 pools across species. Table A.4 performs parallel analysis separately for each species. Its Panels A to D correspond to columns 1 to 4 of Table 1, respectively. Columns 1 and 2 show mean welfare gains and losses, respectively, at the $3 incentive. Column 3 shows the maximum weight $\underline{\alpha}$ that can be placed on those who lose from the transaction while still keeping the transaction admissible at $3. Each panel's estimates are far below the utilitarian weight $\alpha = 0.5$ for each species. Columns 1 and 2 show mean welfare gains and losses, respectively, at

Table A.3: Effect of subjects' ethnicity on participation and reservation prices

VARIABLES	(1) Offer accepted	(2) Reservation price before handout	(3) Reservation price after handout
Ethnicity			
Black	0.035	2.098	1.957
	(0.067)	(4.633)	(4.982)
Asian	-0.061	5.538*	3.247
	(0.039)	(2.955)	(3.189)
Hispanic	0.083	-2.945	-4.300
	(0.070)	(4.898)	(4.993)
Indian	-0.123	15.375**	17.552**
	(0.081)	(6.529)	(7.062)
Other	0.005	1.059	2.190
	(0.074)	(5.170)	(5.988)
High incentive	0.283***		
	(0.032)		
Constant	0.386***	24.865***	28.988***
	(0.028)	(1.557)	(1.738)
Observations	3,276	3,276	3,276
Subjects	671	671	671

Notes: Column 1 estimated by OLS. Columns 2 and 3 estimated with interval regression. White is the omitted category. Standard errors in parentheses, clustered by subject.

the \$30 incentive. Column 6 shows the minimum weight that needs to be placed on those who lose from the transaction to keep the transaction inadmissible at \$30. The estimated thresholds are far above $\underline{\alpha}$ in each case. Hence, the condition $\underline{\alpha} < \overline{\alpha}$ is violated for each species (t-tests, $p < 0.001$ in each case), contrary to UIH-normative. These results are particularly strong in panels B and D, which use ridge regression to average out elicitation error of reservation prices. In both panels, predicted mean welfare gains at the \$3 incentive are 0 for each species.

One potential critique of Experiment 1 concerns the fact that subjects in this experiment have no opportunity to express whether they derive positive utility from eating insects and if so, by how much. Considering non-monetary benefits from the transaction is especially important in light of the position, common in the undue inducement literature, that individuals should be allowed to participate in transactions only if they are intrinsically motivated[2]. To address this point, I estimate the smallest utility benefit that subjects would need to derive from eating insects such that UIH-normative is satisfied ($\underline{\alpha} \leq \overline{\alpha}$). Let Δ denote the amount by which $g(m)$ needs to increase to satisfy the con-

Table A.4: Welfare effects by species in Experiment 1

Variable	(1) $g(\underline{m})$	(2) $l(\underline{m})$	(3) $\overline{\alpha}$	(4) $g(\overline{m})$	(5) $l(\overline{m})$	(6) $\underline{\alpha}$	(7) UIH-normative satisfied	(8) UIH-normative p-value
A. Within-treatment benchmark								
2 house crickets	0.310***	-1.914**	0.139***	13.596***	-1.699**	0.889***	No	0.000
	(0.056)	(0.821)	(0.057)	(0.845)	(0.775)	(0.047)		
5 superworms	0.254***	-1.011**	0.201***	10.372***	-0.325**	0.970***	No	0.000
	(0.050)	(0.495)	(0.086)	(0.865)	(0.153)	(0.014)		
3 silkworm pupae	0.269***	-2.691***	0.091***	12.248***	-2.330***	0.840***	No	0.000
	(0.051)	(0.903)	(0.033)	(0.847)	(0.872)	(0.053)		
2 mole crickets	0.239***	-4.554***	0.050***	11.869***	-9.202***	0.563***	No	0.000
	(0.050)	(1.313)	(0.018)	(0.842)	(2.038)	(0.062)		
2 field crickets	0.358***	-2.803***	0.113***	10.570***	-1.483**	0.877***	No	0.000
	(0.060)	(1.032)	(0.042)	(0.833)	(0.679)	(0.051)		
B. Within-treatment benchmark, predicted by ridge regression								
2 house crickets	0.000	-2.979***	0.000***	13.781***	-2.321***	0.856***	No	0.000
	(0.556)	(0.724)	(0.187)	(0.548)	(0.713)	(0.039)		
5 superworms	0.000	-2.439***	0.000***	9.550***	-0.159	0.984***	No	0.000
	(0.479)	(0.322)	(0.196)	(0.472)	(0.317)	(0.032)		
3 silkworm pupae	0.000	-4.409***	0.000***	10.945***	-3.368***	0.765***	No	0.000
	(0.497)	(0.836)	(0.113)	(0.489)	(0.823)	(0.046)		
2 mole crickets	0.000	-6.993***	0.000***	8.384***	-5.535***	0.602***	No	0.000
	(0.429)	(1.037)	(0.061)	(0.422)	(1.022)	(0.049)		
2 field crickets	-0.000	-3.227***	-0.000***	11.204***	-2.040***	0.846***	No	0.000
	(0.589)	(0.715)	(0.182)	(0.580)	(0.704)	(0.046)		
C. No Video, $3 incentive benchmark								
2 house crickets	0.363***	-2.218***	0.141***	12.940***	-5.039***	0.720***	No	0.000
	(0.060)	(0.860)	(0.052)	(0.873)	(1.388)	(0.061)		
5 superworms	0.312***	-1.571**	0.166***	10.038***	-3.307***	0.752***	No	0.000
	(0.058)	(0.611)	(0.061)	(0.867)	(1.078)	(0.066)		
3 silkworm pupae	0.327***	-3.441***	0.087***	11.063***	-10.169***	0.521***	No	0.000
	(0.058)	(1.045)	(0.029)	(0.860)	(2.227)	(0.063)		
2 mole crickets	0.266***	-5.659***	0.045***	9.607***	-31.724***	0.232***	No	0.000
	(0.053)	(1.539)	(0.015)	(0.832)	(4.515)	(0.035)		
2 field crickets	0.404***	-1.331***	0.233***	12.831***	-0.251	0.981***	No	0.000
	(0.064)	(0.438)	(0.068)	(0.887)	(0.144)	(0.011)		
D. No Video, $3 incentive benchmark, predicted by ridge regression								
2 house crickets	-0.000	-3.827***	-0.000***	9.049***	-4.077***	0.689***	No	0.000
	(0.499)	(0.785)	(0.131)	(0.492)	(0.773)	(0.045)		
5 superworms	-0.000	-3.917***	-0.000***	5.052***	-1.821***	0.735***	No	0.000
	(0.353)	(0.517)	(0.090)	(0.347)	(0.509)	(0.058)		
3 silkworm pupae	0.000	-5.269***	0.000***	7.042***	-5.446***	0.564***	No	0.000
	(0.428)	(0.901)	(0.081)	(0.422)	(0.888)	(0.046)		
2 mole crickets	-0.000	-8.758***	-0.000***	3.190***	-10.344***	0.236***	No	0.000
	(0.260)	(1.168)	(0.030)	(0.256)	(1.151)	(0.028)		
2 field crickets	0.000	-2.367***	0.000***	10.974***	-0.130	0.988***	No	0.000
	(0.529)	(0.273)	(0.224)	(0.522)	(0.269)	(0.024)		

Notes: Each row corresponds to a separate SUR-regression. Columns 1 and 2 show mean welfare gains and losses, respectively, at the $3 incentive. Column 3 shows the maximal weight $\overline{\alpha}$ that may be placed on those who lose from the transaction such the transaction is permissible at the $3 incentive. Columns 4 and 5 show mean welfare gains and losses, respectively, at the $30 incentive. Column 6 shows the minimal weight $\underline{\alpha}$ that must be placed on those who lose from the transaction such that the transaction is inadmissible at the $30 incentive. Reported significance levels in columns 3 and 6 reflect the two-sided z-tests that $\overline{\alpha} = 1$ and $\underline{\alpha} = 0$, respectively. Column 7 indicates whether $\overline{\alpha} \geq \underline{\alpha}$, a necessary condition for UIH-normative. Column 8 lists p-values for two-sided z-tests of the Null hypothesis that $\overline{\alpha} = \underline{\alpha}$. Standard errors in parentheses, clustered by subject. Standard errors in columns 3, 6, and 8 are calculated by the Delta method.

dition. By random assignment of subjects to incentive conditions, Δ is independent of m. Based on the estimates of columns (1), (3), or (4), UIH-normative cannot be satisfied regardless of potential utility benefits because $l(3) < l(30)$ and $g(3) < g(30)$ imply that $W_\alpha(3) < W_\alpha(30)$ for any $\Delta \geq 0$ and any $\alpha \in [0, 1]$. This argument does not apply, however, to the estimates in column (2), where $l(3) > l(30)$. In this case, the minimum increase required to satisfy UIH-normative is $\Delta = \frac{g(30)l(3) - g(3)l(30)}{l(30) - l(3)} = 7.67$. This average utility benefit must be generated by the 4.3 percent of observations in which a subject agreed to eat the insects for free. Hence in these cases, subjects would need to derive the implausibly high utility of at least \$7.67/0.043 = \$178.37 from bug-eating to satisfy UIH-normative.

B SI Experiment 2

Replication of Experiment 2 in Extension Experiment Figure B.1 compares results from Experiment 2 and the Extension Experiment. For comparability, I only use data corresponding to the 50% prior without delayed consequences in this comparison. Panels A and B plot information choice. Panels C and D show posterior beliefs. Panels E and F plot distribution functions of individual welfare. Information choice patterns appear similar across the experiments. So do posterior belief distributions. In both cases, higher incentives cause more optimistic posterior beliefs. The latter effect has two possible causes. First, subjects might have different biases when interpreting information from the Bold Advisor than from the Cautious Advisor. Second, the high incentive might affect how subjects interpret recommendations from a given advisor. Data from the Advisor Choice First treatment suggests that the first mechanism almost entirely drives the increase in posteriors.

Critically, the welfare distributions are also highly similar across the experiment. If anything, choice mistakes increase less when the stakes are larger.

These comparisons indicate that the effects in my experiment are driven by the magnitude of the incentive relative to the potential loss, which is approximately constant across the two panels, rather than by the stake size, which varies by an order of magnitude.

UIH-normative: Additional results I next study the effects of the various treatments on UIH-normative. Panel A of Table B.1 replicates Table 2 from the main text and adds information about mean gains and losses at $\underline{m} = 20$ and $\overline{m} = 80$. Panel B shows the corresponding results for the Extension Experiment with $\underline{m} = 1$ and $\overline{m} = 5$. Column 7 reveals that UIH-normative is violated in eleven of twelve treatments. In five of them, the violation is statistically significant at the 5% level. In all these cases, optimal policy demands that the transaction either be allowed only at sufficiently high incentives or not at all, depending on α. In the former case, a policy that allows the transaction at low but not high incentives *minimizes* welfare.

While the necessary condition for UIH-normative is satisfied in one treatment (regular stakes, contemporaneous, Large Loss, with a 50% prior success probability), the test of the hypothesis that $\underline{\alpha} = \overline{\alpha}$ cannot be rejected at any conventional significance level. Even taking the estimates at face value, UIH-normative is satisfied only for welfare weights α in the narrow range from 0.568 to 0.608. Moreover, once I account for noisy elicitation of certainty equivalents, UIH-normative is violated in this condition, too (see Table B.2).

How do the welfare effects vary across individual treatments? Column 3 shows that a

lower prior success probability leads to substantially tighter upper bounds on the weight that can be placed on losses while keeping the transaction admissible at the low incentive. In fact, with a 20% prior success probability, welfare losses exceed gains by so much that even a utilitarian ($\alpha = 0.5$) prefers preventing the transaction to allowing it at the lowest incentive no matter the remaining conditions. Column 6 shows that a lower prior success probability also increases losses relative to gains at the highest incentive. The Large Loss condition also tends to raise losses relative to gains, compared to the Limited Loss condition. Timing of potential negative consequences does not exert clear and systematic welfare effects. Importantly, none of these level effects translate into support for UIH-normative.

Noisy elicitation of certainty equivalents might bias the estimates in Table B.1 because it causes some misclassification of gains as losses and vice versa. To correct for this possibility, I run ridge regressions with 10-fold cross-validation to predict individuals' certainty equivalents for each of the 26 lotteries they face in stage 2 of the experiment. (The choice of ridge regression is informed by previous research on predicting choice under risk.[3]) I run a separate regression for each round of stage 2. As predictors, I include the gain and loss amounts, the success probability, and a treatment indicator for the Delay condition of the current round. I include all two- and three-way interactions between these variables. Additionally, I include the certainty equivalents of all other rounds, as well as gain and loss amounts, success probabilities, and Delay condition indicators. In addition, I include all two-, three-, and four-way interactions between these predictor variables.

Table B.2 displays the results. The Undue Inducement Condition is violated in all cases but one. The exception occurs in the case of contemporaneous consequences with a prior success probability of 20% in the Large Loss condition. The range of welfare parameters α for which capping incentives is welfare-optimal is given by $[0.153, 0.177]$, which requires the planner to place more than five times as much weight on gains than on losses. By contrast, the undue inducement literature places more weight on individuals who are harmed. According to such welfare weights, the transaction in the affected row should be prevented at all incentives.

Table B.1 tests UIH-normative in an ex-ante welfare framework. Table B.3 reproduces Table B.1 using ex-post welfare, setting w_i equal to the realized monetary gain or loss for each subject in each trial. UIH-normative is violated in all but one cases. Arguably, this is an even stronger result than that based on ex-ante analysis. The reason is that,

in contrast to the ex-ante framework, the ex-post benchmark lets the planner disagree with the subjects' preferences. The violation of UIH-normative according to the ex-post benchmark is particularly noteworthy in the Delay condition, in which the planner imposes judgment not only on risk preferences (by weighting outcomes in different states) but also on time preferences. The violation of UIH-normative in the ex-post framework occurs for a planner who insists that future consequences should not be discounted at all. (In fact, the average subject in the Delay condition is indifferent between a €1 loss incurred with a three-month delay and a €0.83 immediate loss.)

Figure B.1: Comparison Experiment 2 and Extension Experiment.

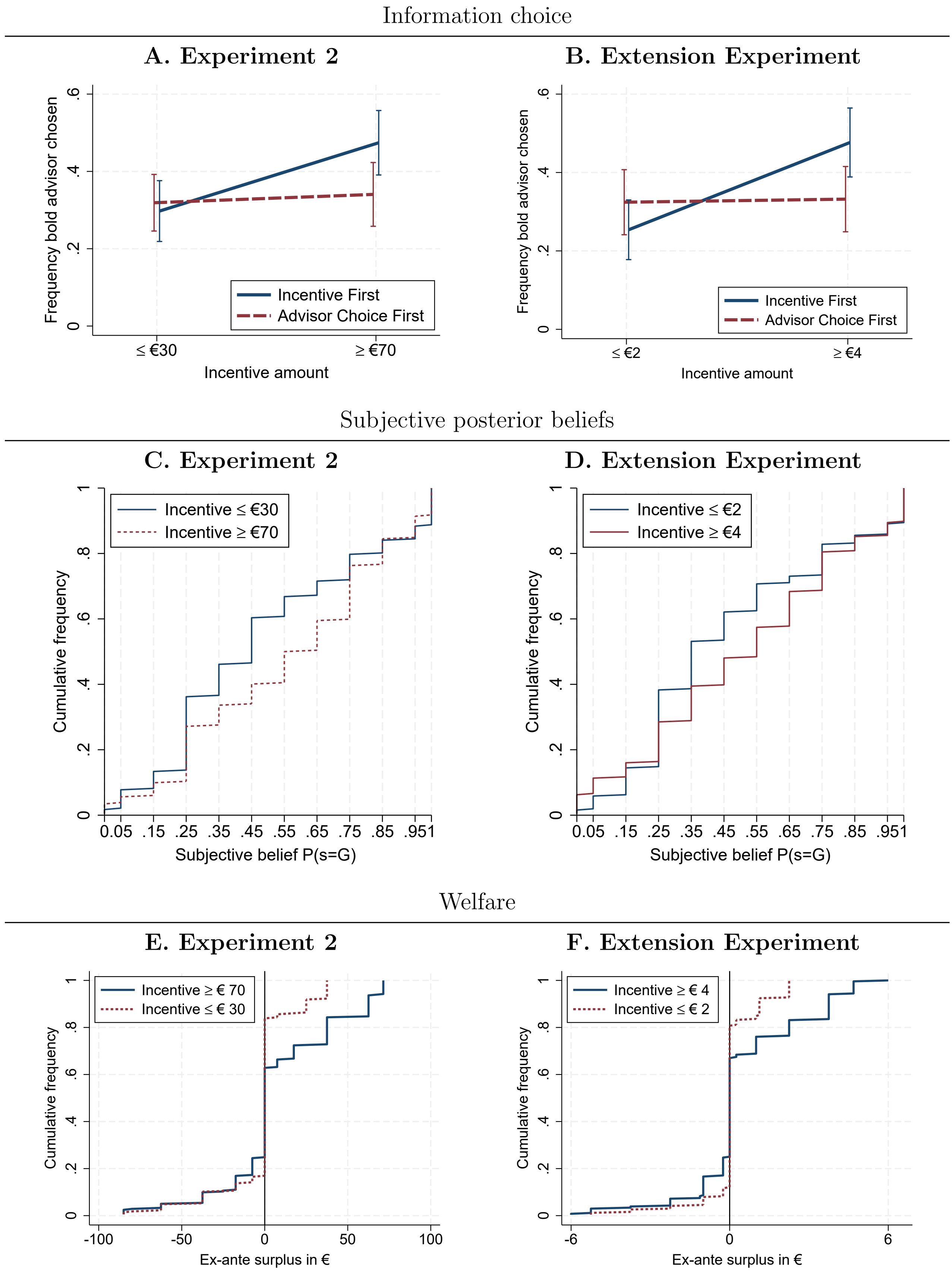

Notes: Panels on the left replicate the corresponding graphs from the main article. To maintain comparability, the panels on the right show the corresponding graphs from the Extension Experiment when the prior risk is 50% and consequences are not delayed. Whiskers in Panels A and B display 95% confidence intervals. Standard errors clustered by subject. Panel A is based on 928 observations from 58 independent subjects. Panel B is based on 5568 observations from 348 independent subjects.

Table B.1: Tests for UIH-normative in Experiment 2 and Extension Experiment

Variable	(1) $g(\underline{m})$	(2) $l(\underline{m})$	(3) $\overline{\alpha}$	(4) $g(\overline{m})$	(5) $l(\overline{m})$	(6) $\underline{\alpha}$	(7) $\underline{\alpha} < \overline{\alpha}$	(8)
							satisfied	p-value
				**A. Experiment 2**				
Loss								
Limited	3.942***	-4.794***	0.451***	19.654***	-4.366**	0.818***	No	0.004
	(1.261)	(1.453)	(0.120)	(2.395)	(1.729)	(0.066)		
Large	4.082***	-6.494***	0.386***	16.125***	-10.532***	0.605***	No	0.084
	(1.065)	(2.196)	(0.106)	(2.508)	(2.185)	(0.068)		
				**B. Extension Experiment**				
Loss								
Limited Loss								
Contemporaneous								
Prior success probability								
20%	0.155***	-0.224***	0.409***	0.357***	-0.290***	0.552***	No	0.395
	(0.059)	(0.083)	(0.138)	(0.092)	(0.087)	(0.080)		
50%	0.100	-0.267**	0.272***	1.315***	-0.167***	0.887***	No	0.001
	(0.070)	(0.105)	(0.165)	(0.185)	(0.045)	(0.034)		
80%	0.267***	-0.121**	0.689	2.468***	-0.113	0.956***	No	0.062
	(0.091)	(0.055)	(0.139)	(0.211)	(0.081)	(0.031)		
Delayed								
Prior success probability								
20%	0.000	-0.207*	0.000***	0.831***	-0.137*	0.858***	No	0.004
	(0.057)	(0.109)	(0.276)	(0.179)	(0.073)	(0.074)		
50%	0.114*	-0.362***	0.239***	1.526***	-0.473***	0.763***	No	0.000
	(0.069)	(0.123)	(0.134)	(0.222)	(0.174)	(0.077)		
80%	0.174**	-0.080**	0.685	2.197***	-0.406**	0.844***	No	0.318
	(0.074)	(0.040)	(0.154)	(0.197)	(0.169)	(0.061)		
Large Loss								
Contemporaneous								
Prior success probability								
20%	0.112*	-0.361**	0.237***	0.359***	-0.501***	0.417***	No	0.397
	(0.064)	(0.170)	(0.143)	(0.121)	(0.134)	(0.114)		
50%	0.175***	-0.113	0.608	0.812***	-0.618***	0.568***	Yes	0.862
	(0.063)	(0.088)	(0.213)	(0.159)	(0.149)	(0.081)		
80%	0.177**	-0.119*	0.597	1.982***	-0.242***	0.891***	No	0.126
	(0.082)	(0.071)	(0.194)	(0.227)	(0.090)	(0.041)		
Delayed								
Prior success probability								
20%	0.069	-0.444***	0.134***	0.324***	-0.283***	0.534***	No	0.016
	(0.046)	(0.161)	(0.091)	(0.108)	(0.096)	(0.128)		
50%	0.168**	-0.237**	0.415***	1.054***	-0.401**	0.724***	No	0.132
	(0.077)	(0.119)	(0.176)	(0.190)	(0.172)	(0.100)		
80%	0.095	-0.159**	0.375***	1.535***	-0.447***	0.774***	No	0.043
	(0.065)	(0.066)	(0.200)	(0.180)	(0.141)	(0.064)		

Notes: Each row corresponds to a separate treatment within the Incentive First condition. Each row reflects a single SUR-regression. Columns 1 and 2 show mean welfare gains and losses, respectively, at the lowest incentive, $\underline{m}$. Column 3 shows the maximal weight $\overline{\alpha}$ that may be placed on those who lose from the transaction such the transaction is permissible at the lowest incentive. Columns 4 and 5 show mean welfare gains and losses, respectively, at the highest incentive, $\overline{m}$. Column 6 shows the minimal weight $\underline{\alpha}$ that must be placed on those who lose from the transaction such that the transaction is inadmissible at the \$30 incentive. Reported significance levels in columns 3 and 6 reflect two-sided z-tests of the Null hypothesis that $\overline{\alpha} = 1$ and $\underline{\alpha} = 0$, respectively. Column 7 indicates whether $\overline{\alpha} \geq \underline{\alpha}$, a necessary condition for UIH-normative. Column 8 lists p-values for two-sided z-tests of the Null hypothesis that $\overline{\alpha} = \underline{\alpha}$. Standard errors in parentheses, clustered by subject. Standard errors in columns 3, 6, and 8 are calculated by the Delta method.

Table B.2: Tests for UIH-normative in Experiment 2 and Extension Experiment controlling for elicitation noise

Variable	(1) $g(\underline{m})$	(2) $l(\underline{m})$	(3) $\overline{\alpha}$	(4) $g(\overline{m})$	(5) $l(\overline{m})$	(6) $\underline{\alpha}$	(7) $\underline{\alpha} < \overline{\alpha}$	(8)
							satisfied	p-value
				**A. Experiment 2**				
Loss								
Limited	2.808***	-3.503***	0.445***	13.517***	-0.890**	0.938***	No	0.000
	(0.456)	(0.987)	(0.085)	(1.440)	(0.404)	(0.028)		
Large	2.135***	-4.465***	0.324***	5.490***	-6.801***	0.447***	No	0.084
	(0.477)	(0.946)	(0.071)	(1.362)	(1.249)	(0.082)		
				**B. Extension Experiment**				
Loss								
Limited Loss								
Contemporaneous								
Prior success probability								
20%	0.084***	-0.191***	0.305***	0.263***	-0.125**	0.678***	No	0.002
	(0.025)	(0.053)	(0.094)	(0.046)	(0.053)	(0.105)		
50%	0.108***	-0.195***	0.356***	0.781***	-0.051**	0.939***	No	0.000
	(0.025)	(0.058)	(0.092)	(0.101)	(0.020)	(0.025)		
80%	0.258***	-0.054**	0.828	2.177***	-0.000	1.000***	No	0.011
	(0.029)	(0.024)	(0.068)	(0.163)	(0.005)	(0.002)		
Delayed								
Prior success probability								
20%	0.040***	-0.141***	0.222***	0.398***	-0.053*	0.883***	No	0.000
	(0.015)	(0.054)	(0.093)	(0.089)	(0.028)	(0.061)		
50%	0.146***	-0.228***	0.391***	0.885***	-0.162	0.845***	No	0.000
	(0.031)	(0.087)	(0.109)	(0.118)	(0.132)	(0.112)		
80%	0.195***	-0.123***	0.614***	1.563***	-0.192*	0.891***	No	0.000
	(0.027)	(0.044)	(0.099)	(0.145)	(0.110)	(0.059)		
Large Loss								
Contemporaneous								
Prior success probability								
20%	0.057***	-0.268***	0.177**	0.089**	-0.494***	0.153**	Yes	0.738
	(0.020)	(0.081)	(0.071)	(0.042)	(0.115)	(0.069)		
50%	0.109***	-0.207***	0.345***	0.281***	-0.376***	0.428***	No	0.403
	(0.026)	(0.061)	(0.092)	(0.065)	(0.094)	(0.092)		
80%	0.202***	-0.065**	0.757***	1.176***	-0.062*	0.950***	No	0.015
	(0.033)	(0.027)	(0.088)	(0.122)	(0.035)	(0.029)		
Delayed								
Prior success probability								
20%	0.027**	-0.288***	0.085**	0.115**	-0.398***	0.224**	No	0.054
	(0.012)	(0.095)	(0.046)	(0.048)	(0.101)	(0.091)		
50%	0.146***	-0.214***	0.406***	0.480***	-0.320**	0.600***	No	0.073
	(0.032)	(0.066)	(0.101)	(0.094)	(0.148)	(0.129)		
80%	0.158***	-0.185***	0.460***	0.894***	-0.154**	0.853***	No	0.000
	(0.032)	(0.055)	(0.098)	(0.126)	(0.071)	(0.064)		

Notes: Each row corresponds to a separate treatment within the Incentive First condition. Each row reflects a single SUR-regression. Columns 1 and 2 show mean welfare gains and losses, respectively, at the lowest incentive, $\underline{m}$. Column 3 shows the maximal weight $\overline{\alpha}$ that may be placed on those who lose from the transaction such the transaction is permissible at the lowest incentive. Columns 4 and 5 show mean welfare gains and losses, respectively, at the highest incentive, $\overline{m}$. Column 6 shows the minimal weight $\underline{\alpha}$ that must be placed on those who lose from the transaction such that the transaction is inadmissible at the \$30 incentive. Reported significance levels in columns 3 and 6 reflect two-sided z-tests of the Null hypothesis that $\overline{\alpha} = 1$ and $\underline{\alpha} = 0$, respectively. Column 7 indicates whether $\overline{\alpha} \geq \underline{\alpha}$, a necessary condition for UIH-normative. Column 8 lists p-values for two-sided z-tests of the Null hypothesis that $\overline{\alpha} = \underline{\alpha}$. Standard errors in parentheses, clustered by subject. Standard errors in columns 3, 6, and 8 are calculated by the Delta method.

Table B.3: Tests for UIH-normative in Experiment 2 and Extension Experiment based on the ex-post welfare benchmark

Variable	(1) $g(\underline{m})$	(2) $l(\underline{m})$	(3) $\overline{\alpha}$	(4) $g(\overline{m})$	(5) $l(\overline{m})$	(6) $\underline{\alpha}$	(7) $\underline{\alpha} < \overline{\alpha}$ satisfied	(8) p-value
	**A. Experiment 2**							
Loss								
Limited	3.876***	-5.272***	0.424***	35.865***	-6.625***	0.844***	No	0.002
	(1.247)	(2.028)	(0.125)	(4.110)	(1.089)	(0.032)		
Large	5.871***	-7.166***	0.450***	31.913***	-17.736***	0.643***	No	0.006
	(0.595)	(1.863)	(0.073)	(3.391)	(3.458)	(0.055)		
	**B. Extension Experiment**							
Loss								
Limited Loss								
Contemporaneous								
Prior success probability								
20%	0.106	-0.517***	0.170***	0.644***	-0.448***	0.590***	No	0.007
	(0.067)	(0.176)	(0.109)	(0.156)	(0.078)	(0.073)		
50%	0.144*	-0.402***	0.264***	1.981***	-0.302***	0.868***	No	0.000
	(0.079)	(0.149)	(0.133)	(0.257)	(0.065)	(0.035)		
80%	0.369***	-0.124	0.749	3.282***	-0.093**	0.972***	No	0.092
	(0.087)	(0.078)	(0.130)	(0.269)	(0.041)	(0.013)		
Delayed								
Prior success probability								
20%	-0.025	-0.397**	-0.066***	0.897***	-0.289***	0.756***	No	0.000
	(0.058)	(0.155)	(0.165)	(0.219)	(0.073)	(0.077)		
50%	0.219***	-0.421**	0.342***	2.091***	-0.309***	0.871***	No	0.000
	(0.082)	(0.170)	(0.121)	(0.274)	(0.070)	(0.032)		
80%	0.190**	-0.227**	0.455***	3.365***	-0.140***	0.960***	No	0.004
	(0.082)	(0.093)	(0.169)	(0.215)	(0.043)	(0.013)		
Large Loss								
Contemporaneous								
Prior success probability								
20%	0.104***	-0.395***	0.208***	0.635***	-0.982***	0.393***	No	0.095
	(0.029)	(0.119)	(0.068)	(0.162)	(0.227)	(0.088)		
50%	0.239***	-0.330***	0.420***	1.787***	-0.779***	0.696***	No	0.008
	(0.034)	(0.108)	(0.084)	(0.223)	(0.188)	(0.062)		
80%	0.466***	-0.050*	0.903	2.962***	-0.619***	0.827***	Yes	0.149
	(0.049)	(0.029)	(0.054)	(0.253)	(0.181)	(0.047)		
Delayed								
Prior success probability								
20%	0.112***	-0.412***	0.214***	0.664***	-0.979***	0.404***	No	0.026
	(0.028)	(0.121)	(0.064)	(0.149)	(0.231)	(0.086)		
50%	0.253***	-0.293***	0.464***	2.112***	-0.896***	0.702***	No	0.017
	(0.039)	(0.107)	(0.105)	(0.239)	(0.250)	(0.070)		
80%	0.353***	-0.109**	0.764	2.746***	-0.440***	0.862***	No	0.358
	(0.041)	(0.055)	(0.094)	(0.226)	(0.144)	(0.043)		

Notes: Each row corresponds to a separate treatment within the Incentive First condition. Each row reflects a single SUR-regression. Columns 1 and 2 show mean welfare gains and losses, respectively, at the lowest incentive, $\underline{m}$. Column 3 shows the maximal weight $\overline{\alpha}$ that may be placed on those who lose from the transaction such the transaction is permissible at the lowest incentive. Columns 4 and 5 show mean welfare gains and losses, respectively, at the highest incentive, $\overline{m}$. Column 6 shows the minimal weight $\underline{\alpha}$ that must be placed on those who lose from the transaction such that the transaction is inadmissible at the $30 incentive. Reported significance levels in columns 3 and 6 reflect the two-sided z-tests of the Null hypothesis that $\overline{\alpha} = 1$ and $\underline{\alpha} = 0$, respectively. Column 7 indicates whether $\overline{\alpha} \geq \underline{\alpha}$, a necessary condition for UIH-normative. Column 8 lists p-values for two-sided z-tests of the Null hypothesis that $\overline{\alpha} = \underline{\alpha}$. Standard errors in parentheses, clustered by subject. Standard errors in columns 3, 6, and 8 are calculated by the Delta method.

C SI Experiment 3

C.1 Design

The experiment in this section follows the same design as Experiment 2, but it provides subjects with an information acquisition technology that allows subjects to access a much wider range of information structures than Experiment 2. Following the paradigm of Dean and Neligh[4], subjects obtain information about whether the venture will be successful by examining a picture of 450 randomly arranged letters as in panel A of Figure C.1. Subjects know that if the venture is a success, the image contains 50 letters G and 40 letters B (for "good" and "bad", respectively) and that these numbers are reversed if the venture is a failure. In contrast to Experiment 2, information costs now depend on the subjects' disutility of effort. Moreover, while subjects in Experiment 2 can, in principle, derive Bayesian posteriors by simple arithmetic, this is not possible in the present experiment. As a result, correct belief updating might be more difficult, worsening choice quality.

Any search strategy induces a probability of participating in the good state and a probability of participating in the bad state, and thus it corresponds to a stochastic information structure. There is no time constraint, so subjects can implement a wide range of search strategies. (Technically, this paradigm is an approximation to the[5] sequential sampling framework, in which any pair of Bayes-consistent posterior beliefs $(\gamma_{\mathscr{G}}, \gamma_{\mathscr{B}}) \in [0,1]^2$ can be implemented as the informativeness of individual draws approaches zero.) They can choose the amount of information to gather in the sense that they have access to information structures that can be Blackwell-ordered. They can also choose the kind of information, for instance, by searching longer before accepting the transaction than before rejecting it. In contrast to Experiment 2, symmetric information structures (in which encouraging and discouraging signals are equally likely ex-ante) are available.

I record subjects' information acquisition as follows. A black panel covers the entire grid of letters, but subjects can use their mouse to move a small hole across the grid through which they can inspect one letter at a time, as in panel B of Figure C.1. I record the sequence of letters the subject observes and use it to calculate Bayesian posteriors. I perform this calculation using the formula $\text{logit}[P(s = G|n, k)] = \text{logit}(\mu) + \log(D)$ where $\text{logit}(x) = \log\left(\frac{x}{1-x}\right)$, where $D = \frac{P(k|n,s=G)}{P(k|n,s=B)} = \frac{(40-k)!}{(50-k)!}\frac{(50-(n-k))!}{(40-(n-k))!}$ is the likelihood ratio for observing n draws of informative letters (G or B) with k letters G, as follows directly from the hypergeometric distribution function.

The set of treatments is the same as in the Extension Experiment. I also use the same parameters, with two exceptions. First, all amounts are in USD instead of EUR. Second, the incentive amounts are \$0.5, \$1, \$5, and \$5.5. I use the term *Grid First* to

Figure C.1: Presentation of information about the state.

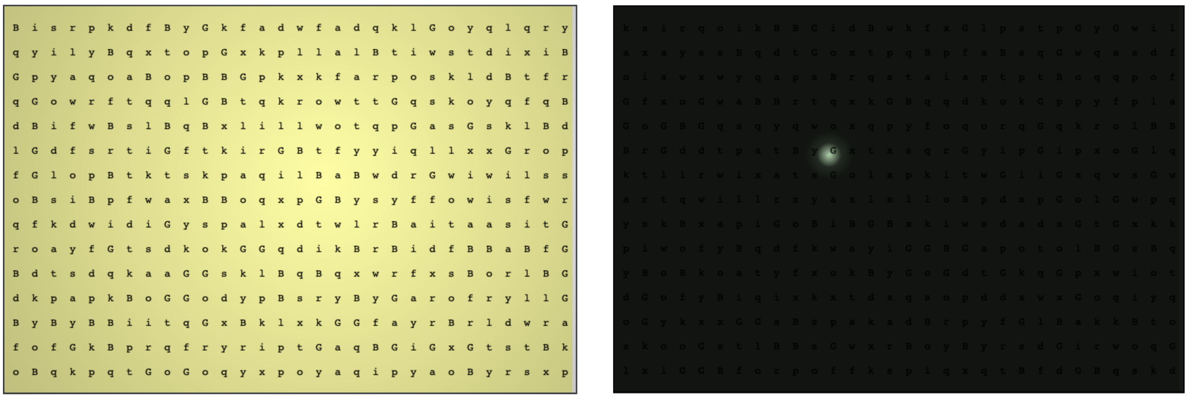

(A) (B)

Notes: Panel A: If the venture is good, the picture has 50 letters G and 40 letters B. If the venture is bad, it has 40 letters G and 50 letters B. Panel B: The subject moves the mouse to change the location of the hole in the black panel.

refer to the case in which subjects examine the grid of letters before learning about the incentive amount for the round (corresponding to the Advisor Choice First condition in Experiment 2).

Because searching a grid of letters is more time-consuming than the choice of advisors in Experiment 2, stage 1 of this experiment consists of only 16 rounds, corresponding to 4 incentive levels, the Incentive First and Grid First condition, and the Limited and Large Loss conditions, administered in random order.

To highlight the independence of the rounds, each picture is presented with a different background color. The color assignment is randomized on the individual level. A state is drawn independently for each subject and decision, and a new picture of randomly scrambled letters is generated. Subjects could not use a text editor to automatically count the letters because they were presented in a picture format (HTML5 Canvas).

Elicitation of the ex-ante welfare benchmark Stage 2 of the current experiment presents subjects with lotteries corresponding to the decisions the subject faced in each round of stage 1 if she updated her beliefs according to Bayes' law. The subject proceeds through 16 such decisions corresponding to stage 1, as well as through 12 additional decisions for which the success probability is exogenously given by 0.2, 0.5, and 0.8, once for each of the incentive amounts \$1 and \$5 and once for each of the Limited and Large Loss conditions, respectively. A total of 28 rounds are administered in individually randomized order.

The survey automatically calculates the Bayesian posterior depending on the letters the subject has uncovered during her search. If a subject rapidly moves the mouse across

the display, letters may be displayed for imperceptibly brief periods. Research shows that subjects cannot identify letters with any accuracy if they are displayed for less than 50 milliseconds.[6] Accordingly, I record a letter as seen only if it is displayed for 50 milliseconds or longer. The Bayesian posteriors subjects encounter in stage 2 of the experiment are calculated based on this benchmark.

Implementation I conducted this experiment on the Amazon Mechanical Turk online labor market with a total of 405 subjects on November 9-12, 2020. I required subjects to be located in the US, have at least a 99% approval rate on previous tasks completed on the platform, and have completed at least 500 tasks on the platform. Each subject receives a \$12 completion payment from which potential losses are discounted. Subjects in the Delay condition receive \$6 of these \$12 with a three-month delay (net of any losses they may incur). In addition, subjects receive a base payment of \$1. Subjects could only participate on a desktop or laptop computer with a screen sufficiently large to display all pictures without scrolling. The median subject completed the experiment in 63.4 minutes; average earnings are \$13.58. Subjects examined each image for a median time of 12 seconds, with 55 seconds at the 75th percentile and 2 seconds at the 25th percentile.

Because the experiment is administered online, attrition is a possible concern. 614 subjects begin the survey on a device that is technically equipped to display the survey. Of these, 70 attrit during the instructions before reaching the first comprehension check. Another 104 do not progress beyond the first comprehension check, and another 11 attrit at or before the second comprehension check. Of these 429 subjects, 405 completed the entire survey. In the instructions, subjects learn whether they will receive some part of the payment with a three-month delay, and they learn the prior success probability they will face. Accordingly, attrition could vary by treatment condition. Amongst the 209 subjects who attrited, 99 were in the Contemporaneous condition, and 110 were in the Delay condition, whereas 75, 81, and 53 faced a 20%, 50%, or 80% prior success probability, respectively. Regressing an indicator for whether a subject attrited on an indicator for the Delay condition, the numerical prior success probability and the interaction between the two have no explanatory power ($p > 0.3$ for separate t-tests on all coefficients except for the constant term, and $p > 0.5$ for the F-test of the joint explanatory power of all variables compared to a constant-only model).

C.2 Analysis

I first examine the effect of incentives on information acquisition. The undue inducement literature is concerned that higher incentives lead to less informed decision-making.

Figure C.2: Results of Experiment 3.

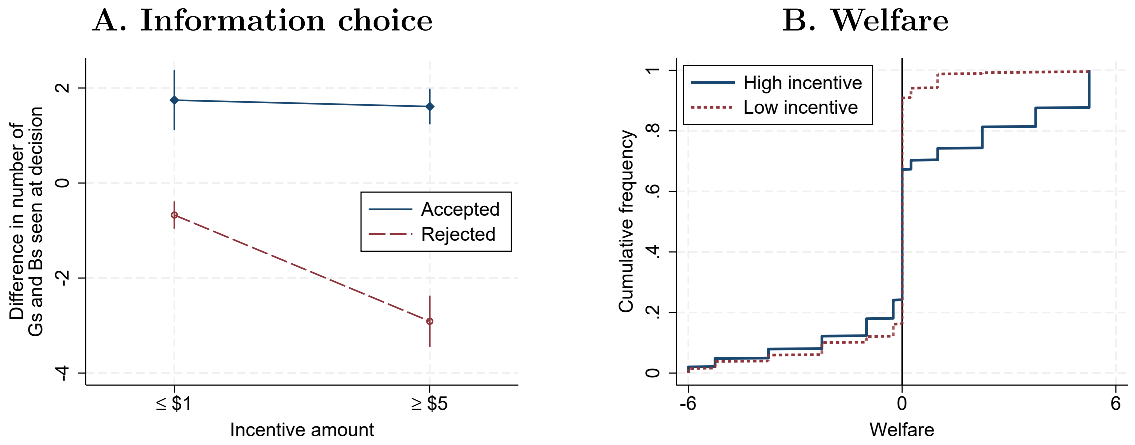

Notes: Panel A: Mean difference between the number of Gs and the number of Bs seen when making a decision. Spikes show 95% confidence intervals. Standard errors are clustered by subject. Based on 6480 observations from 405 independent subjects. Panel B: Ex-ante welfare. For visibility, I group the $0.5 and $1 incentives into the low incentive category and the $5 and $5.5 incentives into the high incentive category.

On the contrary, higher incentives lead to increased search in the Incentive First condition (pooling across all remaining conditions), as measured by the number of informative letters ("B" or "G") in the string of letters the subject observed. The magnitude of the effect is substantial. In the case of the $0.5 incentive, subjects parse strings with an average of 12.4 informative letters. This number nearly doubles to 22.6 informative letters for the case of the $5.5 incentive ($p < 0.001$ for the difference). Hence, on average, higher incentives do not cause less careful decision-making.

Yet, incentives might still cause subjects to alter their information acquisition in a way reminiscent of UIH-positive. UIH-positive predicts that subjects will consult information they more likely expect to recommend participation when the incentive is higher. In the present experiment, a subject who seeks to increase the chance of observing a string of data that implies the risk is less likely to realize than the prior probability (i.e., a string that contains more Gs than Bs), for instance, can achieve by selecting a stronger evidentiary standard before rejecting the transaction than before rejecting it. Specifically, she can accept the transaction as soon as she has seen just a few more letters G than B, but reject only if she has seen many more letters B than G. Previous research investigates these mechanisms formally[7].

Panel A of Figure C.2 shows that incentives do exert this effect. I plot how many more Gs than Bs a subject has observed before making a participation decision, separately for acceptance and rejection, by incentive amount. The evidentiary standard subjects require for accepting the transaction is to observe just under two more Gs than Bs regardless

of the incentive. The evidentiary standard for rejecting the transaction becomes much stricter when the incentive is high, as one would expect, given that mistakenly rejecting the transaction implies a larger foregone gain when the incentive is higher ($p < 0.001$). This behavior is reminiscent of UIH-positive, though the larger amount of information acquired in the high-incentive case does not suggest that higher incentives would decrease decision quality.

To test UIH-normative, I use the same methods as in Experiment 2. Panel B of Figure C.2 displays the welfare distribution for the Incentive First, Large Loss condition, pooling across the remaining conditions. (Certainty equivalents were not recorded for the case of Limited Loss in the Incentive First condition.) Just as in Experiments 1 and 2, higher incentives greatly increase the average welfare gain and have little, if any, effect on the average welfare loss.

Table C.1 performs formal tests analogous to Table B.1. As column 7 shows, UIH-normative is violated throughout.

Overall, this experiment demonstrates that the conclusions of Experiment 2 are robust to a more flexible form of information acquisition.

D SI Theory

Setting A Bayesian expected utility maximizer decides whether or not to participate in a transaction in exchange for a material incentive m. The consequences of participation depend on a state of the world s, which is good ($s = G$) with prior probability μ, and bad ($s = B$) otherwise. If the agent participates and the state is good, he experiences utility $u_G(m) > 0$. If the agent participates and the state is bad, he experiences disutility $u_B(m) < 0$. If the agent abstains, he receives no payment and experiences utility 0. I assume that $u_G(m)$ is strictly increasing in m and that $u_B(m)$ is weakly increasing in m, reflecting the fact that higher compensation never decreases a participants' utility in a given state (though, in principle, it may lower his ex-ante expected utility by causing him to participate more often in the bad state). The functions $u_G(m)$ and $u_B(m)$ encompass the agent's risk preferences.

Before deciding whether to participate in the transaction, the agent chooses an information structure that produces a stochastic signal about the state. (An information structure I is a function $I : \{G, B\} \to \Delta\mathscr{S}$ that maps states of the world into probability distributions over a space of signals $\mathscr{S}$.) Critically, I assume that the agent's information processing capacity is limited, so he needs to be selective about the kind of information to consult. Formally, the agent chooses an information structure from a finite set

Table C.1: Welfare effects in Experiment 3

Variable	(1) $g(\underline{m})$	(2) $l(\underline{m})$	(3) $\overline{\alpha}$	(4) $g(\overline{m})$	(5) $l(\overline{m})$	(6) $\underline{\alpha}$	(7) UIH-normative satisfied	(8) UIH-normative p-value
Loss								
Limited (Grid First)								
Contemporaneous								
Prior success probability								
20%	0.018	-0.500***	0.035***	0.359***	-1.684***	0.176***	No	0.034
	(0.017)	(0.144)	(0.032)	(0.107)	(0.234)	(0.054)		
50%	0.019	-0.547***	0.034***	0.863***	-0.801***	0.518***	No	0.000
	(0.022)	(0.150)	(0.039)	(0.157)	(0.137)	(0.073)		
80%	-0.013	-0.221**	-0.064***	2.257***	-0.322***	0.875***	No	0.000
	(0.033)	(0.090)	(0.170)	(0.238)	(0.110)	(0.044)		
Delayed								
Prior success probability								
20%	0.043	-0.315***	0.119***	0.325**	-0.935***	0.258***	No	0.307
	(0.038)	(0.113)	(0.103)	(0.134)	(0.213)	(0.097)		
50%	0.020	-0.273**	0.070***	0.955***	-0.789***	0.548***	No	0.007
	(0.044)	(0.119)	(0.143)	(0.155)	(0.162)	(0.074)		
80%	0.037	-0.413***	0.082***	2.240***	-0.371***	0.858***	No	0.000
	(0.050)	(0.143)	(0.102)	(0.242)	(0.135)	(0.051)		
Large (Incentive First)								
Contemporaneous								
Prior success probability								
20%	0.021	-0.462***	0.044***	0.408***	-0.841***	0.327***	No	0.008
	(0.024)	(0.148)	(0.050)	(0.126)	(0.180)	(0.089)		
50%	0.024	-0.387***	0.058***	0.736***	-0.763***	0.491***	No	0.000
	(0.035)	(0.116)	(0.083)	(0.143)	(0.157)	(0.078)		
80%	0.036	-0.261**	0.122***	2.100***	-0.314**	0.870***	No	0.000
	(0.057)	(0.106)	(0.175)	(0.263)	(0.124)	(0.051)		
Delayed								
Prior success probability								
20%	0.032	-0.668***	0.046***	0.482***	-0.661***	0.422***	No	0.000
	(0.033)	(0.224)	(0.048)	(0.159)	(0.199)	(0.113)		
50%	0.069	-0.230**	0.232***	0.882***	-0.533***	0.623***	No	0.009
	(0.050)	(0.094)	(0.150)	(0.166)	(0.146)	(0.086)		
80%	0.081	-0.434***	0.157***	2.574***	-0.238**	0.915***	No	0.000
	(0.050)	(0.122)	(0.080)	(0.251)	(0.114)	(0.040)		

Notes: Each row corresponds to a separate treatment. Each row reflects a single SUR-regression. Columns 1 and 2 show mean welfare gains and losses, respectively, at the lowest incentive, $\underline{m}$. Column 3 shows the maximal weight $\overline{\alpha}$ that may be placed on those who lose from the transaction such the transaction is permissible at the lowest incentive. Columns 4 and 5 show mean welfare gains and losses, respectively, at the highest incentive, $\overline{m}$. Column 6 shows the minimal weight $\underline{\alpha}$ that must be placed on those who lose from the transaction such that the transaction is inadmissible at the $30 incentive. Reported significance levels in columns 3 and 6 reflect two-sided z-tests of the Null hypothesis that $\overline{\alpha} = 1$ and $\underline{\alpha} = 0$, respectively. Column 7 indicates whether $\overline{\alpha} \geq \underline{\alpha}$, a necessary condition for UIH-normative. Column 8 lists p-values for two-sided z-tests of the Null hypothesis that $\overline{\alpha} = \underline{\alpha}$. Standard errors in parentheses, clustered by subject. Standard errors in columns 3, 6, and 8 are calculated by the Delta method.

$\mathscr{I} = \{I_1, \ldots, I_n\}$ with $n \geq 2$ which may represent, for instance, advisors with various biases. For simplicity, I assume each information structure's signal space has two elements, $\mathscr{G}$ and $\mathscr{B}$, which I interpret as a recommendation to participate and a recommendation to abstain, respectively. I let p_I^s denote the probability that information structure I produces signal $\mathscr{G}$ if the state is s. Without loss of generality, I assume $p_I^G \geq p_I^B$ for each information structure $I \in \mathscr{I}$.

I will show that participation incentives change the kind of information a rational agent demands, specifically, the optimal information structure's *bias*, as defined in Gentzkow et al.[8]. In my setting, that definition specializes as follows:

Definition 1. Consider two information structures I and I' and a given prior μ. Then, I' is *biased towards* $\mathscr{G}$ relative to I if $P(I' = \mathscr{G}) \geq P(I = \mathscr{G})$. Moreover, I' is *statewise biased towards* $\mathscr{G}$ relative to I if $p_{I'}^s \geq p_I^s$ for all states $s \in \{G, B\}$.

Analysis The agent's optimization problem involves two stages. First, the agent selects an information structure. Second, he decides whether to participate based on his observed signal realization. The selected information structure is *instrumental* if, in the second stage, the agent chooses to participate after observing signal realization $\mathscr{G}$ and chooses to abstain after observing signal realization $\mathscr{B}$. The agent also has the opportunity to ignore the signal realization and to either participate or abstain with certainty. I account for this possibility by defining the information structures $I^{\mathscr{G}} = (1, 1)$ and $I^{\mathscr{B}} = (0, 0)$, and letting $\bar{\mathscr{I}} = \mathscr{I} \cup \{I^{\mathscr{G}}, I^{\mathscr{B}}\}$. Considering the agent's choice from $\bar{\mathscr{I}}$, I can then assume, without loss of generality, that the agent follows the signal produced by the chosen information structure. Thus, the agent's probability of participating in state s equals the probability of a good signal in that state, p_I^s. Because the agent's utility from non-participation is 0, his utility maximization problem in the first stage is

$$\max_{I \in \bar{\mathscr{I}}} \mu p_I^G u_G(m) + (1 - \mu) p_I^B u_B(m). \tag{1}$$

The main result of this section shows that Bayes-optimal behavior appears as if the agent engaged in *motivated reasoning*: as the incentive m increases, the agent chooses an information structure with a stronger bias towards $\mathscr{G}$. (Formally, the solution to (1), which may not be unique, is increasing in m in the strong set order.) Intuitively, this result arises from a simple cost-benefit calculation. Information helps the agent limit choices that are *ex-post mistaken* in the sense that the chosen action is suboptimal conditional on the state of the world (even though the choice may have been optimal conditional on the information available at the time of decision). There are two possible ex-post mistakes.

A *false positive* occurs if the agent participates in the bad state. A *false negative* occurs if the agent abstains in the good state. The agent seeks to minimize the cost of these mistakes, which depends on the incentive amount m. A higher incentive decreases the cost of false positives (because the additional money acts as insurance against ex-post undesirable outcomes) and increases the cost of false negatives (because abstention means foregoing a larger amount of money). Accordingly, as the incentive increases, the agent becomes more interested in preventing false negatives and less interested in preventing false positives. The agent achieves this goal by selecting an information structure more strongly biased towards $\mathscr{G}$.

Proposition 1. *Consider incentive amounts m and m' with $m > m'$. Suppose at least one information structure is instrumental at m, and at least one information structure is instrumental at m'. Let I^* and I'^* denote the solutions to (1) at m and m', respectively, and suppose that they are unique and $I^* \neq I'^*$. Then I^* is statewise more biased towards $\mathscr{G}$ than I'^*.*

Proof. To prove the claim, I parametrize the constraint, insert it into the objective function, and characterize the optimal information structure. I then show how a change in m changes the optimal information structure.

The objective function (1) is linear in (p_G, p_B), with indifference curves that are upward-sloping in (p_G, p_B)-space. Accordingly, the only candidates for optimal information structures lie on the boundary of the free-disposal convex hull H of the set of information structures. Formally, H is defined as follows. A point (p_G, p_B) is in H if and only if there exists a subset of information structures $(I_1, ..., I_n) \subseteq \tilde{\mathscr{I}}$ with weights $a_1, ..., a_n$ such that $\sum_{k=1}^{n} a_k p_I^G \geq p_G$ and $\sum_{k=1}^{n} a_k (1 - p_I^B) \geq (1 - p_B)$.

Let $\mathscr{J} \subseteq \tilde{\mathscr{I}}$ denote the subset of information structures on the boundary of H; let n denote the cardinality of $\mathscr{J}$. Enumerate these information structures such that $p_{I_1}^G \leq p_{I_2}^G \leq ... \leq p_{I_n}^G$. Because H is convex, the boundary of H is convex. Formally, $\frac{p_{I_k}^B - p_{I_{k-1}}^B}{p_{I_k}^G - p_{I_{k-1}}^G}$ is an increasing function of k for $2 \leq k \leq n$.

The boundary of H implicitly defines p_B as a function of p_G. This function, denoted $b(p_G)$, is piecewise linear and continuous. Accordingly, we can write the objective function in (1) as $u(p_G; m) = \mu p_H u_G(m) + (1 - \mu) b(p_G) u_B(m)$. A composition of a finite number of continuous, piecewise linear functions, $u(p_G; m)$ is continuous and piecewise linear. For each $k = 2, ..., n$ the slope of $m(p_G; m)$ as a function of p_G over the interval $(p_{I_{k-1}}^G, p_{I_k}^G)$ is

given by

$$u'(p_G; m) = \mu u_G(m) + (1-\mu)\frac{p^B_{I_k} - p^B_{I_{k-1}}}{p^G_{I_k} - p^G_{I_{k-1}}} u_B(m)$$

Because $u_B(m) < 0$ and because $\frac{p^B_{I_k} - p^B_{I_{k-1}}}{p^G_{I_k} - p^G_{I_{k-1}}}$ is increasing, $u'(p_G)$ is decreasing.

Let k^* denote the optimal information structure. If $u'(p_G; m) > 0$ for all $p_G \in (p^G_{I_1}, p^G_{I_n})$, the optimal information structure is I_n, hence $k^* = n$. If $u'(p_G; m) < 0$ for all $p_G \in (p^G_{I_1}, p^G_{I_n})$, the optimal information structure is I_1, hence $k^* = 1$. Otherwise, there exists $\hat{k} = 2, ..., n-1$ such that $u'(p_G; m)$ is weakly positive to the left of $p^G_{I_{k^*}}$ and weakly negative to the right. In that case, $\hat{k}$ is within the set of optimal information structures. If both inequalities are strict, then $k^* = \hat{k}$. If $u'(p_G, m)$ is zero to the left of $p^G_{I_{k^*}}$, then the set of optimal information structures includes $\hat{k} - 1$. If $u'(p_G, m)$ is zero to the right of $p^G_{I_{k^*}}$, then the set of optimal information structures includes $\hat{k} + 1$.

How does an increase in m affect the optimal information structure? Consider m', which, as assumed in the proposition, satisfies $m' > m$. Define $k^*(m')$ as the index k such that $u'(p_G; m')$ is positive to the left of $p^G_{I_{k^*(m')}}$ and negative to the right if such a value exists; otherwise set $k^*(m') = 1$ if $u'(p_G; m')$ is uniformly negative and set $k^*(m') = n$ if $u'(p_G; m')$ is uniformly positive. Then, $k^*(m')$ is the optimal information structure at incentive m'. Because $u_G(m)$ and $u_B(m)$ are both increasing in m, an increase in m to $m' > m$ increases $u'(p_G; m)$. Accordingly, $k^*(m') \geq k^*(m)$ in the strong set order.

Finally, the assumption that both m and m' are associated with at least one information structure that is instrumental implies that neither $k^*(m')$ nor $k^*(m)$ are equal to the unconditional participation or abstention information structures $(0, 0)$ or $(1, 1)$. This completes the proof. □

Figure D.1 illustrates Proposition 1 graphically. It plots an example of a set of information structures $\mathscr{I}$ in $(p_G, 1-p_B)$-space. These information structures, together with unconditional participation and abstention information structures $I^{\mathscr{G}}$ and $I^{\mathscr{B}}$, constitute the agent's choice set $\bar{\mathscr{I}}$. The agent's utility increases to the northeast. By expected utility, the agent's indifference curves are straight lines in $(p_G, 1-p_B)$-space. Therefore, it suffices to consider the (free disposal) convex hull of the agent's opportunity set, shaded in gray in the figure. A higher incentive m increases the benefit of participation in the good state, p_G, and (weakly) decreases the benefit of abstention in the bad state, 1 - p_B. Accordingly, an increase in m tilts indifference curves counterclockwise. Therefore, the optimal information structure associated with a higher incentive lies further to the north-

Figure D.1: Bayesian information choice

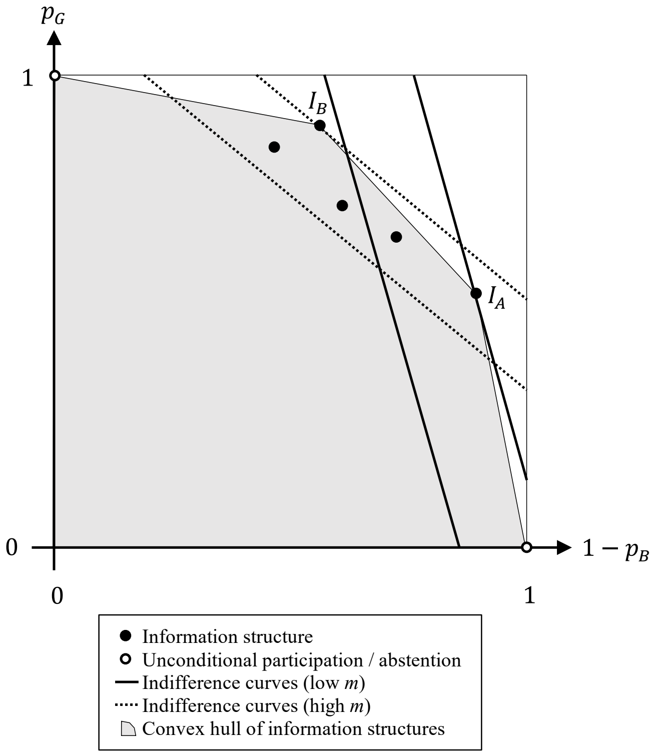

west. In the figure, the optimal information structure for a given low incentive amount is labeled A, and the optimal information structure for a given high incentive amount is labeled B. The remaining information structures lie in the interior of the convex hull of the agent's opportunity set. Hence, they will never be chosen.

Welfare While a rational agent will never participate in a transaction at an ex-ante welfare loss, boundedly rational individuals may make such mistakes, and higher incentives may increase their frequency, as I show by example. Hence, for non-Bayesians, behavior that satisfies UIH-positive may facilitate UIH-normative. Specifically, consider the family of welfare functions that place weight α on losses and weight $(1-\alpha)$ on gains, along with the set of information structures $\mathscr{I} = \{I^{\mathscr{G}}, I^{\mathscr{B}}\}$ with $I^{\mathscr{G}} = (1, 0.5)$ and $I^{\mathscr{B}} = (0.5, 0)$ (the parameters of Experiment 2). Hence, $I^{\mathscr{G}}$ only recommends abstention if the state is bad, and $I^{\mathscr{B}}$ only recommends participation if the state is good. Consider two incentive levels $\underline{m}$ and $\overline{m}$ with $0 < \underline{m} < \overline{m}$. Suppose an agent chooses $I^{\mathscr{B}}$ in case of incentive $\underline{m}$, chooses $I^{\mathscr{G}}$ in case of incentive $\overline{m}$ (consistent with UIH-positive), and always follows the signal produced by the chosen information structure, possibly due to excess optimism upon receiving a signal $\mathscr{G}$. In the case of the low incentive, $m = \underline{m}$, this agent never participates in the bad state but sometimes participates in the good state. Accordingly, at $\underline{m}$, mean welfare gains are positive, $E\big(g(\underline{m})\big) > 0$, and mean welfare losses are zero,

$E\big(l(\underline{m})\big) = 0$, both ex-ante and ex-post, implying $\overline{\alpha} = 1$. At incentive $\overline{m}$, by contrast, there is a positive probability that the agent participates in the bad state and suffers an ex-post loss. In the case of ex-post analysis, this fact directly implies $E\big(l(\overline{m})\big) < 0$, and thus $\underline{\alpha} < 1$, so that UIH-normative is satisfied. Notably, this is true even if the agent is Bayesian and chooses information rationally because ex-post welfare analysis permits the planner's weight on losses to differ from the agent's risk attitudes. In the case of ex-ante welfare $E\big(l(\overline{m})\big) < 0$ obtains if the subject is non-Bayesian (making it possible to observe the assumed participation strategy) and sufficiently risk-averse (placing sufficient weight on the potential loss). In this case, too, $\underline{\alpha} < 1$, so UIH-normative is satisfied.

References

[1] Maggie E Toplak, Richard F West, and Keith E Stanovich. Assessing miserly information processing: An expansion of the cognitive reflection test. *Thinking & Reasoning*, 20(2):147–168, 2014.

[2] Alex John London. Undue inducements and reasonable risks: will the dismal science lead to dismal research ethics? *The American Journal of Bioethics*, 5(5):29–32, 2005.

[3] Alexander Peysakhovich and Jeffrey Naecker. Using methods from machine learning to evaluate behavioral models of choice under risk and ambiguity. *Journal of Economic Behavior & Organization*, 133:373–384, 2017.

[4] Mark Dean and Nathaniel Neligh. Experimental tests of rational inattention. *Journal of Political Economy*, forthcoming.

[5] Abraham Wald. *Sequential Analysis*. New York: Wiley, 1947.

[6] Claus Bundesen and Lisbeth Harms. Single-letter recognition as a function of exposure duration. *Psychological Research*, 62(4):275–279, 1999.

[7] Stephen Morris and Philipp Strack. The wald problem and the equivalence of sequential sampling and static information costs. *Available at SSRN: https://ssrn.com/abstract=2991567*, 2019.

[8] Matthew Gentzkow, Jesse M Shapiro, and Daniel F Stone. Media bias in the marketplace: Theory. In *Handbook of media economics*, volume 1, pages 623–645. Elsevier, 2015.
