# Supplementary material for: An experimental test of whether financial incentives constitute undue inducement in decision-making
Source: Nat Hum Behav. 2024 Mar 8;8(5):835–45. doi: 10.1038/s41562-024-01817-8 (PMC11132984; doi:10.1038/s41562-024-01817-8)
Supplement: Supplementary file 2 — Reporting Summary [file 41562_2024_1817_MOESM2_ESM.pdf]

nature portfolio

Corresponding author(s): Sandro Ambuehl

Last updated by author(s): Nov 8, 2023

Reporting Summary

Nature Portfolio wishes to improve the reproducibility of the work that we publish. This form provides structure for consistency and transparency in reporting. For further information on Nature Portfolio policies, see our Editorial Policies and the Editorial Policy Checklist.

Statistics

For all statistical analyses, confirm that the following items are present in the figure legend, table legend, main text, or Methods section.

n/a	Confirmed	
☐	☒	The exact sample size (*n*) for each experimental group/condition, given as a discrete number and unit of measurement
☐	☒	A statement on whether measurements were taken from distinct samples or whether the same sample was measured repeatedly
☐	☒	The statistical test(s) used AND whether they are one- or two-sided<br>*Only common tests should be described solely by name; describe more complex techniques in the Methods section.*
☐	☒	A description of all covariates tested
☐	☒	A description of any assumptions or corrections, such as tests of normality and adjustment for multiple comparisons
☐	☒	A full description of the statistical parameters including central tendency (e.g. means) or other basic estimates (e.g. regression coefficient) AND variation (e.g. standard deviation) or associated estimates of uncertainty (e.g. confidence intervals)
☐	☒	For null hypothesis testing, the test statistic (e.g. *F*, *t*, *r*) with confidence intervals, effect sizes, degrees of freedom and *P* value noted<br>*Give P values as exact values whenever suitable.*
☒	☐	For Bayesian analysis, information on the choice of priors and Markov chain Monte Carlo settings
☒	☐	For hierarchical and complex designs, identification of the appropriate level for tests and full reporting of outcomes
☒	☐	Estimates of effect sizes (e.g. Cohen's *d*, Pearson's *r*), indicating how they were calculated

Our web collection on statistics for biologists contains articles on many of the points above.

Software and code

Policy information about availability of computer code

Data collection	All data in this study were collected with the Qualtrics Research Core survey tool.
Data analysis	All analysis in this study was done using Stata/MP 16.0 for Mac (64-bit Intel)

For manuscripts utilizing custom algorithms or software that are central to the research but not yet described in published literature, software must be made available to editors and reviewers. We strongly encourage code deposition in a community repository (e.g. GitHub). See the Nature Portfolio guidelines for submitting code & software for further information.

Data

Policy information about availability of data

All manuscripts must include a data availability statement. This statement should provide the following information, where applicable:

- Accession codes, unique identifiers, or web links for publicly available datasets
- A description of any restrictions on data availability
- For clinical datasets or third party data, please ensure that the statement adheres to our policy

The datasets generated during and analyzed during the current study are available in a replication package on the Harvard Dataverse, https://doi.org/10.7910/DVN/3PFZKP.

Human research participants

Policy information about studies involving human research participants and Sex and Gender in Research.

Reporting on sex and gender	All analyses pool across sexes / genders. The terms "sex" and "gender" do not appear in the manuscript. Appendix table B.1 lists the fraction of (self-reported) male participants across treatments in Experiment 1. Following established standards in experimental economics, I do not perform gender-based analysis. Based on concerns about forking paths and fishing for statistical significance, these standards advise researchers not to perform such subgroup analyses unless the are a main focus of the study design which is not the case in the present study.
Population characteristics	Participants in Experiment 1 were undergraduate students at Stanford University, the Ohio State University, and the University of Michigan. Of the total of 671 subjects, 313 were women, 353 were men, and 5 were nonbinary. Mean age was 21.56 years. Participants in Experiment 2 and the Extension Experiment were undergraduate students at the University of Cologne. Of the 58 subjects who completed this experiment, 38 were women and 20 were men, with a mean age of 27.05. Of the 348 subjects who completed the Extension Experiment, 208 were women, and 140 were men, with a mean age of 26.63.
Recruitment	Recruitment was done by sending invitation emails to the subject pools of the experimental economics laboratories at each university where a session was run. Selection into the study was limited by the fact that invitation emails did not reveal any study details. Assignment to treatment was randomized across (experiments 1 and 2) as well as within (experiment 2) subject. Treatment effect estimates are unbiased for the study population due to random assignment to treatment.
Ethics oversight	Experiment was approved by Stanford University's Non-medical IRB in protocol #34001. Experiment 2 was approved by the University of Zurich in protocol OEC IRB # 2022-007.

Note that full information on the approval of the study protocol must also be provided in the manuscript.

Field-specific reporting

Please select the one below that is the best fit for your research. If you are not sure, read the appropriate sections before making your selection.

☐ Life sciences ☒ Behavioural & social sciences ☐ Ecological, evolutionary & environmental sciences

For a reference copy of the document with all sections, see nature.com/documents/nr-reporting-summary-flat.pdf

Behavioural & social sciences study design

All studies must disclose on these points even when the disclosure is negative.

Study description	The experiments in this study follow the standards of experimental economics. Human subjects make decisions that have consequences for the payment they will receive for participation and for any other activities they will have to perform after the study. Measurement is quantitative.
Research sample	Experiment 1 uses US undergraduate students at three universities. The study sample was chosen following standards in experimental economics (which typically use such subject pools) as well as due to the need for physical interaction with participants. The sample is not representative of the general US population. Of the total of 671 subjects, 313 were women, 353 were men, and 5 were nonbinary. Mean age was 21.56 years. Experiment 2 and the Extension Experiment use German university students in Cologne who participated online. The study sample was chosen following standards in experimental economics during the Covid19-pandemic that precluded in-person studies. The sample is not representative of the general German population. Of the 58 subjects who completed this experiment, 38 were women and 20 were men, with a mean age of 27.05. Of the 348 subjects who completed the Extension Experiment, 208 were women, and 140 were men, with a mean age of 26.63.
Sampling strategy	Samples for both experiments are convenience samples. The laboratories at which the experiments were conducted maintain databases of subjects interested in participation who were invited by email. For experiment 1, Power simulations revealed that on the order of 800 subjects would be required, though due to unexpectedly low subject availability at the University of Michigan a sample of 671 participants was ultimately obtained. For experiment 2, sample size was determined based on the results of a pilot experiment conducted at the University of Toronto (which included only one of the two main outcome measures).
Data collection	All data in both experiments were collected with the Qualtrics Research Core survey tool. In experiment 1, only the researcher was present in the experimental sessions. Between 10 and 30 subjects participated in each session and made decisions on individual computer terminals. All subjects in a given session either participated in the video condition or in the no-video condition. Hence, the researcher was not blind to those treatments. Assignment to incentive condition was randomized by the computer on the subject level in each session, rendering the experimenter blind to this treatment assignment. In experiment 2, subjects participated online

and assignment to treatment was randomized by the computer, rendering the experimenter blind to treatment assignment.

Timing	Data for experiment 1 were collected in 39 sessions in May, June, and July 2015. Data for Experiment 2 were collected on February 7, 2022. Data for the Extension Experiment were collected November 25 to 27, 2020.
Data exclusions	No data were excluded from analysis.
Non-participation	No participants dropped out or declined participation in experiment 1. In Experiment 2, there was no attrition. In the Extension Experiment, the 348 complete responses make up 95.6\% of the 364 surveys that were started on a machine that satisfied the technical requirements (desktop or laptop computers with sufficient screen width). Out of the 16 attriters, 3 did not proceed to the first comprehension check, 10 dropped out at the first comprehension check, 1 dropped out at the second comprehension check, and 2 left the survey after the second comprehension check. Attrition is unrelated to treatment. The variation of incentive amounts and information order occur within subject. Hence, the corresponding main treatment estimates are unaffected by differential attrition.
Randomization	Partcipants were randomly assigned to treatment by the Qualtrics survey in both experiment 1 and experiment 2. Assignment to video condition in experiment 1 occurred at the session level.

Reporting for specific materials, systems and methods

We require information from authors about some types of materials, experimental systems and methods used in many studies. Here, indicate whether each material, system or method listed is relevant to your study. If you are not sure if a list item applies to your research, read the appropriate section before selecting a response.

Materials & experimental systems

n/a	Involved in the study	
☒	☐	Antibodies
☒	☐	Eukaryotic cell lines
☒	☐	Palaeontology and archaeology
☒	☐	Animals and other organisms
☒	☐	Clinical data
☒	☐	Dual use research of concern

Methods

n/a	Involved in the study	
☒	☐	ChIP-seq
☒	☐	Flow cytometry
☒	☐	MRI-based neuroimaging
